# Targeted siRNA delivery reduces nitric oxide mediated cell death after spinal cord injury

**DOI:** 10.1186/s12951-017-0272-7

**Published:** 2017-05-08

**Authors:** Wen Gao, Jianming Li

**Affiliations:** Department of Basic Medical Sciences, Center for Paralysis Research, College of Veterinary Medicine, Purdue University, 408 S. University St., West Lafayette, IN 47907 USA

**Keywords:** siRNA, Chitosan nanoparticles, Spinal cord injury, M1 macrophage, iNOS, Apoptosis

## Abstract

**Background:**

Traumatic spinal cord injury (SCI) includes the primary insult as well as a sequela of biochemical and cellular cascades that amplifies the initial injury. This degenerative process, known as secondary injury, is often mediated by both reactive oxygen and nitrogen species released from damaged cells. Previous data suggests that dysregulated production of nitric oxide via inducible nitric oxide synthase (iNOS) is detrimental to spinal cord recovery. M1 macrophages have been implicated to overexpress iNOS post-SCI. In this work, we propose to inhibit iNOS expression through small interfering RNA (siRNA) complexed chitosan nanoparticles (NPs) that primarily target M1 macrophages.

**Methods:**

siRNA conjugated chitosan complexes were fabricated with and without an antibody (Ab) targeting moiety and screened for efficiency to reduce iNOS expression in vitro. Best formulations were subsequently applied in vivo following acute SCI in a rodent model. iNOS expression as well as Bax and Bcl-2 biomarkers were used to assess cell apoptosis within the lesion at 24 h post-injury.

**Results:**

Ab-siRNA conjugated chitosan NPs significantly reduced iNOS expression in vitro in M1 polarized macrophages. Results show high transfection efficiency with low cytotoxicity. Subsequent application of NPs in vivo after SCI demonstrated both a reduction in iNOS expression and cellular apoptosis.

**Conclusion:**

Proof of concept indicates that siRNA conjugated chitosan NPs can downregulate iNOS production and inhibit apoptosis following SCI. Our proposed gene silencing method putatively targets M1 macrophages as a means to attenuate secondary injury.

**Electronic supplementary material:**

The online version of this article (doi:10.1186/s12951-017-0272-7) contains supplementary material, which is available to authorized users.

## Background

Following spinal cord injury (SCI), a number of cellular and biochemical events are initiated that exacerbate the primary insult. This secondary injury is often associated with dysregulated production of reactive oxygen and nitrogen species (ROS/RNS). These highly unstable compounds overwhelm the endogenous antioxidant system and perpetuate a vicious cycle of cellular bystander damage [1]. The propagation of this oxidative stress can also damage proteins, DNA and membrane phospholipids [2, 3]. Long-lasting participants in this inflammatory process include pro-inflammatory M1 macrophages, which along with the traditional role of debridement, can secrete toxins that induce neuronal cell death [4, 5]. Activated M1 macrophages are known to produce excessive levels of nitric oxide (NO), superoxide, and cytotoxic molecules peroxynitrite (ONOO^−^) due to the synergistic enzymatic activity of inducible nitric oxide synthase (iNOS) within the first few days after SCI [6]. The upregulation of iNOS and its synthesized free radical nitric oxide has deleterious effects on neurons and have been further implicated in neuroinflammation and chronic wounds [7–10]. Evidence shows iNOS deficient mice demonstrate improved functional recovery following spinal cord damage [11] while the application of iNOS antisense oligonucleotides have also increased the number surviving neurons after SCI [12]. Therefore, iNOS appears to be a viable therapeutic target in the acute phase of SCI.

Recently, small interfering RNA (siRNA) has emerged as a promising tool to control gene expression in many pathological conditions that include various cancers, genetic disorders, immune system diseases, and infections [13–15]. Several target proteins have been investigated with siRNA for administration following SCI. These include the inhibition of anti-regenerative proteins RhoA and ERK2 [16–18]. However, there are many practical challenges that remain towards developing siRNA therapeutics for the clinic. Cellular penetration of naked siRNA is poor due to its size and polarity, and rapid enzymatic degradation of the nucleic acids can be problematic. Design of appropriate carriers is therefore essential for optimal transfection efficiency. Chitosan, a linear biocompatible polysaccharide, has shown potential in biomedical applications as both a scaffolding material and delivery vehicle. With siRNA delivery, the cationic nature of chitosan in acidic environments can be exploited to prevent the negatively charged siRNAs from degrading and to facilitate cellular transfection [19, 20]. For instance, expansion of the polymer from charge effects or osmotic swelling of the endosome via protonation of the chitosan amine groups can lead to physical rupture of the endosome during vesicle-mediated endocytosis [21, 22]. Chitosan can be further decorated with other moieties to promote siRNA escape from the endosome or to improve cellular specificity [20, 22]. Studies have also noted that the chitosan polymer can limit lipid peroxidation and mitigate secondary injury after primary SCI [23]. Thus, chitosan NPs have both a physiochemical and biologic merit for possible implementation into SCI therapeutics.

In this work, our goal was to develop siRNA-chitosan complexes as a means to attenuate iNOS expression in M1 macrophages post-SCI. The proposed NPs were conjugated with antibodies to facilitate Fc-receptor mediated phagocytosis by M1 polarized macrophages in vitro. Proof of concept studies using the NPs were conducted in vitro, first to characterize the physical stability of the siRNA-chitosan complex, and secondly, to assess gene silencing and transfection efficiency. Exemplary formulations were subsequently applied to the mouse spinal cord following a clinically relevant compression injury scheme. Biomarkers used to characterize cell apoptosis related secondary injury included expression of iNOS and the Bcl-2 family of proteins. The experimental results provide a basis for using siRNA NPs for moderating the early stage immune response after spinal cord trauma.

## Methods

### Synthesis of siRNA conjugated chitosan NPs

Two different types of siRNA-chitosan NPs were prepared (i) siRNA-chitosan NPs and (ii) siRNA-chitosan NPs with an antibody (Ab) targeting moiety. For siRNA-chitosan, the synthesis was as follows: Low molecular weight chitosan (MO, Sigma-Aldrich) was dissolved in 0.2 M sodium acetate buffer (pH 4.5) and adjusted to a pH of 5.5 with sodium hydroxide. 1 ml of chitosan solution was prepared at a concentration of 200 μg/ml. Then, 400 nM iNOS-siRNAs were added to 1 ml of chitosan solution while stirring. The solutions were then kept at room temperature for 1 h. For the Ab-siRNA-chitosan NPs, 270 μg/ml purified mouse IgG solution was mixed with 200 μg/ml chitosan solution. Then, 0.2 mM of 1-ethyl-3-[3-dimethylaminopropyl]carbodiimide hydrochloride (EDC) (MA, Thermo Fisher Scientific) and 0.5 mM *N*-hydroxysulfosuccinimide (sulfo-NHS) (MA, Thermo Fisher Scientific) were added to the antibody modified chitosan mixture as a crosslinking agent. The solutions were then stirred for 24 h at 4 °C. The following day, the solutions were transferred into 10 K MWCO slide-A-Lyzer Dialysis Cassettes (MA, Thermo Fisher Scientific) and stirred for 48 h to remove undesired particulates <10,000 daltons. Different ratios of IgG modified chitosan solution at 10, 20 or 30% v/v were separately conjugated with 200 μg/ml chitosan solution. Then, 400 nM of the siRNA solution was added to each of these chitosan solutions and stirred. The Ab-siRNA-chitosan NPs were kept at room temperature for 1 h before use.

### Transmission electron microscopy

For electron microscopy, one drop of chitosan nanoparticle solution was first deposited onto mesh copper grids coated with amorphous carbon and allowed to settle at room temperature for 2 min. Negative staining was subsequently performed by swishing through 2% uranyl acetate droplet before imaging. All samples were imaged using a FEI Tecnai T20 transmission electron microscope operating at 200 kV.

### Size and surface charge of NPs

The size and zeta potential of the NPs were analyzed using a Zetasizer Nano ZS (UK, Malvern). Both size and zeta potential were measured in triplicate using distilled water as a dispersant. Measurements were set at 13° + 173° angle and a temperature of 25 °C. The sample solutions were then measured for particle size and zeta potential. The z-average hydrodynamic diameter was calculated based on the viscosity and refractive index of water. All dynamic light scattering and zeta potential results were analyzed with supplied Malvern software.

### Gel retardation assay

A displacement assay using poly(l-aspartic acid) (PAA) (TX, Santa Cruz Biotechnology) was conducted to detect the integrity of siRNA conjugated chitosan nanocomplexes. PAA was used to displace the negatively charged siRNA from the positively charged chitosan. NPs were incubated with or without PAA at concentration of 5 mg/ml. A 1:4 volumetric ratio between PAA and the nanocomplexes was administered and subsequently incubated at 37 °C for 30 min. 50 μl of sample solutions containing 0.5 μg of siRNA were then placed into wells of 10% Mini-protean TBE polyacrylamide gels (CA, Bio-Rad). Electrophoresis was then performed at 100 V for 100 min. The gel was then transferred to SYBR gold nucleic acid staining solution (MA, Thermo Fisher Scientific) for 30 min and visualized using GBox imager.

### Differentiation of M1 and M2 macrophages

Bone marrow-derived macrophages (BMDM) were obtained from the tibias and femurs of common C57BL/6 adult mice (IL, Cell Biologics, Inc.). The cell culture medium was prepared using Dulbecco’s modified Eagle medium/F12, 10 mM l-glutamine and supplemented with 10% fetal bovine serum, 100 U/ml penicillin, and 100 μg/ml streptomycin. Macrophages were seeded in 12- well or 24-well tissue culture plates at 1 × 10^6^ cells and allowed to adhere for 24 h at 37 °C in humidified atmosphere containing 5% CO_2_ prior to experimental studies. Macrophages were polarized to the M1 state by incubating with cell medium containing 5 ng/ml granulocyte–macrophage colony-stimulating factor **(**GM-CSF) and stimulated with a combination of 100 ng/ml lipopolysaccharides (LPS) (MO, Sigma-Aldrich) and 20 ng/ml interferon γ (IFNγ) (CA, B&D Bioscience) for 12 h. On the other hand, macrophages were incubated with 2500 U/ml Macrophage colony-stimulating factor (M-CSF) and treated with 20 ng/ml interleukin 4 (IL-4) (CA, eBioscience) for 12 h to activate into the M2 state. The control group was the macrophages incubated with GM-CSF without any cytokine stimulation. The morphology of macrophages was imaged using Olympus IX 81 inverted fluorescent microscope.

### Toxicity studies

Toxicity of siRNA delivered with lipid transfection reagent (siRNA w/TR) and siRNA-chitosan NP variants were assessed based on morphological changes to the macrophages. Macrophages were cultured in a 24-well plate as previously described. Cells at each well were incubated with 40 nM siRNA combining with Lipofectamine 2000 transfection reagent (MA, Thermo Fisher Scientific) or siRNA-chitosan NPs with or without antibody for 4 h. Macrophages were then observed with an Olympus IX 81 inverted microscope in brightfield mode.

### In vitro siRNA screening

The iNOS target siRNA sequences used were as follows: GAAACGUUAUCAUGAAGAU (siRNA1), CAUGGGAGCCACAGCAAUA (siRNA2), GGAGAUGGUCCGCAAGAGA (siRNA3) and GAUUUAGAGUCUUGGUGAA (siRNA4) (CO, GE Dharmacon). Prior to activation into the M1 state with 100 ng/ml LPS and 20 ng/ml IFNγ, macrophages cultured in serum free medium were treated with 40 nM siRNA w/TR per well for 4 or 48 h. The other two groups of macrophages separately received the siRNA-chitosan NPs or Ab-siRNA-chitosan NPs treatment for 4 h. For all experiments, cells were incubated in fresh medium for 12 h between treatment and macrophage activation. For comparison, other negative control groups containing scrambled siRNA were also prepared to measure iNOS expression.

Macrophages activated via exposure to LPS and IFNγ were then incubated in ice-cold RIPA buffer (MA, Thermo Fisher Scientific) and kept on ice for 5 min. Macrophage lysates were gently collected using cell mini scraper and centrifuged for 15 min at 19,000×*g* at 4 °C. The supernatant was quantified by BCA protein assay kit (MA, Thermo Fisher Scientific), with equal amounts of the proteins from different samples loaded into 7.5% sodium dodecyl sulfate (SDS)-polyacrylamide gels and transferred to a polyvinylidene difluoride (PVDF) membrane. Membranes were incubated in 1× Tris Buffered Saline blocking buffer with Tween^®^ 20 (TBST, MA, Cell Signaling Technology) and 5% w/v nonfat dry milk for 1 h at room temperature. Immunoblotting was processed through incubating membranes with mouse specific primary antibody iNOS (MA, Cell Signaling Technology) and β-Actin Antibody (MA, Cell Signaling Technology) overnight at 4 °C. The next day, the membranes were gently agitated in blocking buffer containing anti-rabbit IgG, HRP-linked Antibody (MA, Cell Signaling Technology) for 1 h at room temperature. Then, LumiGLO solution was prepared by diluting 20× LumiGLO and 20× peroxide (MA, Cell Signaling Technology) in 1× miniQ water. Lastly, membranes were developed in LumiGLO solution for 1 min at room temperature. The bands of interest were detected by Syngene GBox imager and analyzed using Image J software.

### Nitric oxide detection

For nitric oxide detection, the macrophage groups that were studied for iNOS expression were also prepared in the same manner. However, another group, naked siRNA without any transfection reagent, was also added for evaluation. Samples were collected and separately detected for nitrate (${\text{NO}}_{3}^{-}$) and nitrite (${\text{NO}}_{2}^{-}$) levels using Nitric Oxide Assay Kit (MA, Thermo Fisher Scientific).

### Transfection efficiency

BMDM were cultured in a 12-well dish at density of 1 × 10^6^. Chitosan NPs were prepared as described above with the exception that DY547 conjugated siRNAs (customized by GE Dharmacon) were used (sense sequences DY547-CAUGGGAGCCACAGCAAUAUU and antisense 5′-PUAUUGCUGUGGCUCCCAUGUU siRNA). The treatment groups of siRNA w/TR and Ab-siRNA-chitosan NPs were cultured for 4 h prior to imaging. The fluorescence signals and macrophage distribution was imaged by Olympus IX 81 inverted microscope.

### Compression model of spinal cord injury

All animal care and treatment were carried out according to the guidelines of the Purdue Animal Care and Use Committee (PACUC). Female BALB/c mice with weight approximate 25 g were used in this study. Spinal cord injury was induced via a compression technique while animals were under isofluorane inhalational anesthesia. During this procedure, all the mice received a bilateral, hemi-laminectomy at low thoracic region. The spinal cord was crushed using compression forceps that deformed the cord to 0.15 mm of the original cord width (~10% of original width). Ab-siRNA-chitosan NPs solution as described above was centrifuged for 15 min at 4 °C. The pellet was then prepared as 10 μg siRNA strands in 10 μl chitosan NPs suspension. All siRNA treatments were conducted by injecting chitosan NPs solution to spinal cord via Hamilton syringe with 30 gauge needles for 10 min. The lesion was surgically closed in layers, and then the skin was closed and sutured. Sham animals also underwent the same surgical procedure except no Ab-siRNA-chitosan NPs were applied.

### Western blot analysis for spinal cord extracts

At 24 h following SCI, the mice were anesthetized and euthanized. The injured spinal cord segments (0.5 cm long) were rapidly removed and frozen in dry ice. Before use, the frozen tissues were massed and placed into vials with high impact zirconium beads (1.5 mm) and lysis solution containing RIPA buffer, 1× Halt Protease and Phosphatase Inhibitor Cocktail, and 1× EDTA (MA, Thermo Fisher Scientific). The samples were then homogenized using BeadBug Microtube Homogenizer (NJ, Benchmark Scientific). The homogenate was centrifuged at 10,000×*g* for 5 mins at 4 °C. The protein concentration of tissue lysates was then determined with a BCA Protein Assay Kit (MA, Thermo Fisher Scientific). 50 µl aliquots were subjected to 4–20% polyacrylamide gel, and the proteins were electrophoretically transferred to PVDF membrane filters. The membrane then was blocked with 5% nonfat milk in TBST for 1 h at room temperature. After washing, the membranes were incubated with primary antibody solution containing iNOS, α-tubulin (1:1000, MA, Cell Signaling Technology), Bcl-2 (1:200), and Bax (1:100) (CA, Santa Cruz Biotechnology) separately overnight at 4 °C. The next day, the membrane was washed 3 times with TBST and probed with HRP conjugated secondary antibody for 1 h at room temperature. This procedure was then followed by the incubation of LumiGLO solution for 1 min at room temperature. The membrane was exposed to a Syngene GBox imager and the density of the immunoreactive bands was quantified using ImageJ software.

### Statistical analysis

All data are shown with standard error of the mean (±SEM). One-way ANOVA along with a post hoc Tukey’s HSC multiple comparisons tests was used via SPSS software. Significance was determined using a p value ≤0.05.

## Results

### Chitosan NPs

**Fig. 1:**
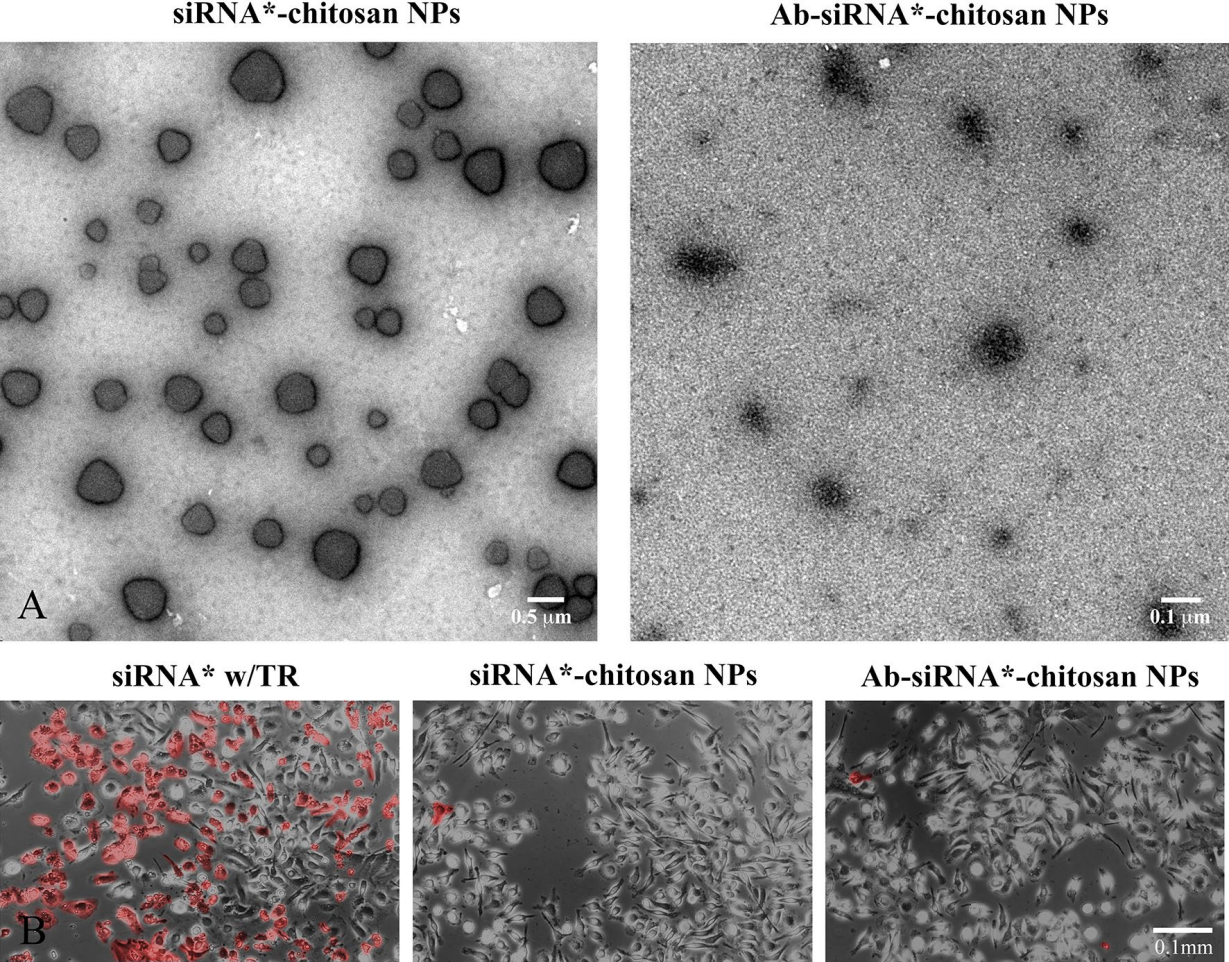
**A** Transmission electron micrographs of different siRNA conjugated chitosan NPs. *Left* siRNA-chitosan NPs, *scale bar* 0.5 μm. *Right* Ab-siRNA-chitosan NPs, *scale* 0.1 μm. **B** Acute cytotoxicity of cells exposed to siRNA delivered with transfection reagent (TR) after a 4 h exposure time. Morphological based cytotoxicity was observed as cytoplasmic vacuolation, granularity or cellular debris (pseudocolored *red*). Macrophages treated with siRNA w/TR showed the highest cell death whereas the chitosan vehicles demonstrated minimal cytotoxicity

The selection and volume of chitosan used during nanoparticle synthesis can lead to a large structural and functional versatility of the NPs. To optimize siRNA conjugated chitosan NPs, the molecular weight (MW), degree of deacetylation (DD), pH, and N/P ratio (or weight ratio) as important criteria were considered in the design of NPs formation (see “Discussion”). The morphologies of two different chitosan NPs are illustrated in Fig. 1 using transmission electron microscopy. The siRNA-chitosan NPs possessed a smooth surface with spherical-like shapes. Ab-siRNA-chitosan NPs showed a rougher and less defined surface due to antibody conjugation. Random fields from 10 micrographs did not reveal particle agglomeration as all NPs were well defined and distinct.

### Particle size and surface charge

Size distribution and surface charge analysis was performed by dynamic light scattering and are shown in Table 1. Mean particle size of siRNA-chitosan NPs was measured to be 272.2 ± 8.13 nm. The size of Ab-siRNA-chitosan NPs with different antibody ratios was also measured and showed that 10% Ab-siRNA-chitosan NPs had an average hydrodynamic diameter 295.53 ± 6.47 nm. The size of NPs with antibody ratios of 20 and 30% v/v were 350.58 ± 9.63, and 385.73 ± 4.78 nm, respectively. Zeta potential analysis revealed that all nanocomplexes were positively charged, having zeta potentials in the range of 17–25 mV at pH 5.5. These results suggest acceptable nanoparticle stability based on electrostatic properties. However, increasing the number of antibody moieties tended to neutralize the zeta potential, with a concurrent increase in particle size. This results show reduced stability for NPs heavily decorated with antibodies.

**Table 1:** Size distribution and surface charge of different siRNA conjugated chitosan NPs

Name	Size (nm)	Zeta potential (mV)
siRNA-chitosan NPs	272.2 ± 8.13	23.63 ± 0.18
10% Ab-siRNA-chitosan NPs	295.53 ± 6.47	24.33 ± 0.73
20% Ab-siRNA-chitosan NPs	350.58 ± 9.63	21.93 ± 0.61
30% Ab-siRNA-chitosan NPs	385.73 ± 4.78	17.77 ± 0.18

### Divergent morphologies of M1 and M2 macrophages

Activation of different polarized macrophages via cytokine induction was highly correlated with a concurrent change in macrophage morphology. Macrophages receiving no cytokine stimulation showed a round or spindly morphology. Induction into the M2-like with the addition of IL-4 resulted in more elongated macrophages. In contrast, macrophages stimulated with a combination of LPS and IFNγ displayed a flattened or circular morphology with outspread cytoplasm (Additional file 1: Figure S1).

### Binding affinity between siRNAs and chitosan NPs

To detect the stability of electrostatic interaction between siRNA strands and chitosan, an electrophoretic gel retardation assay was performed. Co-administration of a strong polyanion PAA was used as a competitor to displace siRNA from the chitosan nanocomplex. In both siRNA chitosan NPs formulations, the addition of PAA resulted in the displacement of siRNA from the chitosan nanocomplex (Additional file 1: Figure S2). In comparison, nanocomplexes not exposed to PAA did not show any sign of free siRNA. This demonstrates protection of the siRNA by the chitosan vehicle. Therefore, absent a strong competitive force, the siRNA strands are not easily separated from the chitosan complex.

### Cytotoxicity of chitosan NPs

BMDM macrophages incubated with siRNA w/TR for 4 h were analyzed for toxicity-associated morphological changes (Fig. 1). In these cells, a significant amount of cytoplasmic vacuolation and granularity was observed. Cells were also more clustered, rounded in appearance with extracellular debris present on the dish bottom. In the contrast, no marked signs of cell deterioration for macrophages treated with the chitosan NPs were observed. Only minimal signs of cellular debris were found. Macrophages retained their original shape and were remained firmly attached.

### Screening of iNOS targeting siRNA

The silencing effects of four different siRNA sequences on iNOS protein expression were evaluated in vitro with M1 polarized macrophages. M2 macrophages and untreated controls were used as a basis for comparison. Negligible iNOS expression was observed in both the control and IL-4 activated M2 macrophage groups. In contrast, there was a significant increase in iNOS expression following M1 macrophage activation (Additional file 1: Figure S3). Of all sequences, both siRNA1 and siRNA*2 (also indicated as siRNA* in later context) showed significant suppression in iNOS compared to activated M1 macrophages that were not exposed to the siRNA treatment (**p ≤ 0.01 and ***p ≤ 0.001, respectively). NO production also confirmed these result (Additional file 1: Figure S5). At a higher concentration of 1 μg/ml LPS and 200 ng/ml IFNγ challenge, M1 macrophages also showed sufficient iNOS attenuation with siRNA*2. As a result, this siRNA sequence was used in all following experiments (Additional file 1: Figure S4).

### Efficient transfection of antibody conjugated siRNA-chitosan NPs

**Fig. 2:**
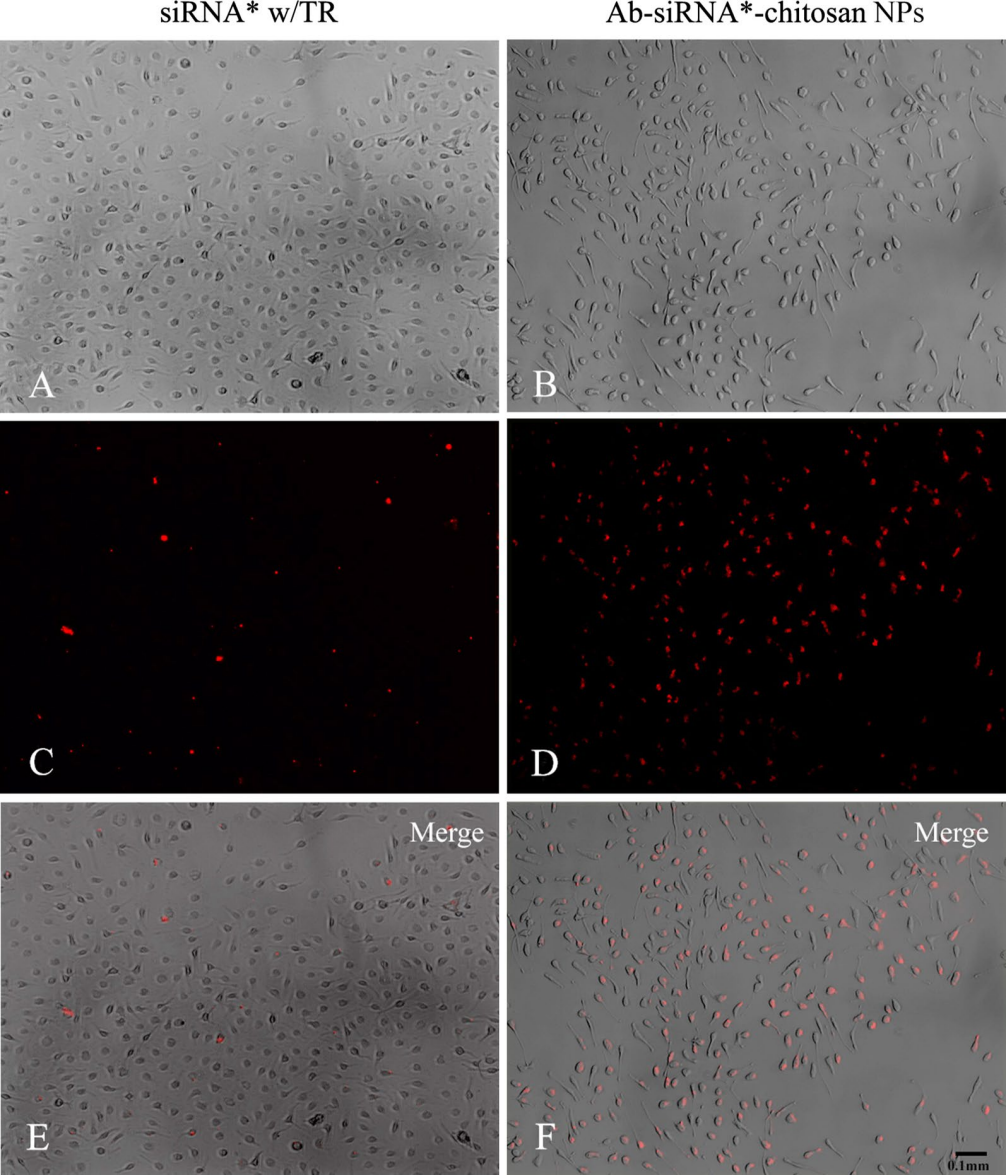
Representative transfection efficiency of siRNA w/TR and 10% Ab-siRNA-chitosan NPs. **A**, **B** Bright field images of cultured macrophages. **C**, **D** Fluorescent images demonstrating uptake of DY547 conjugated siRNA per delivery vehicle. **E**, **F** Merged images depicting proportion of siRNA uptake

The intracellular distributions of siRNA w/TR and Ab-siRNA-chitosan NPs in the murine BMDM were studied to evaluate the efficiency of siRNA transfection. Customized DY547 dye conjugated with the 5′end of siRNA strand was used to visualize the cellular uptake. DY547 signals, which denote siRNA or siRNA chitosan nanocomplexes, were imaged using fluorescence microscopy and expressed as a red fluorescence signal (Fig. 2). Macrophage population was imaged in bright field mode and the images merged with the fluorescence signals. The results visually outline the transfection efficiency when using either the transfection reagent or the Ab coupled chitosan NPs in facilitating siRNA delivery. The internalization of Ab-siRNA-chitosan NPs was dramatically higher (~60 to 70%) when compared to the results with the lipid based transfection agent (~10 to 20%) at a 4 h incubation time.

### Reduction of iNOS expression in vitro

**Fig. 3:**
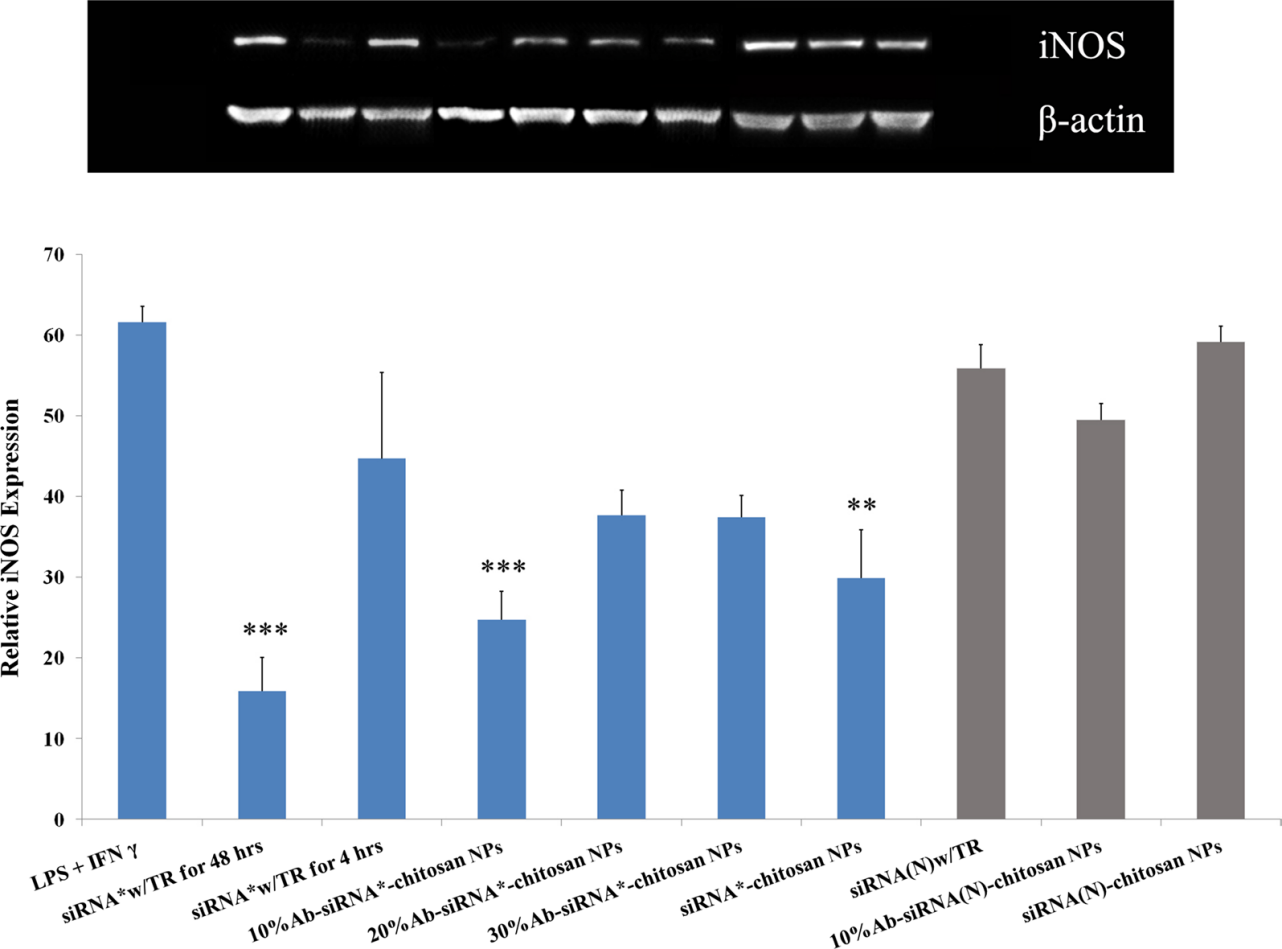
Knockdown of iNOS mRNA in activated M1 macrophages. All groups were challenged with 100 ng/ml LPS and 20 ng/ml IFNγ for 12 h. Activated macrophages without siRNA silencing were indicated as LPS + IFNγ. siRNA* w/TR for 48 h denotes cells transfected with siRNA and a lipid transfection reagent for 48 h. Stimulated cells were also exposed to siRNA w/TR for 4 h (siRNA* w/TR for 4 h). Different antibody conjugated chitosan NPs ranging from 10 to 30% Ab level were also screened for efficacy in lowering iNOS expression (indicated as 10, 20, 30% Ab-siRNA*-chitosan NPs, respectively). siRNA conjugated chitosan NPs without the antibody moiety are labeled as siRNA*-chitosan NPs. Chitosan NPs coupled to scrambled siRNA sequences [siRNA (N)] either with or without antibody conjugation are denoted with* grey bars*. **p ≤ 0.01; ***p ≤ 0.001 vs LPS + IFNγ

Changes in iNOS expressions were first assessed with siRNA pre-treatment groups delivered via transfection reagents at 48 or 4 h. At 4 h exposure, no statistical significance was found for siRNA w/TR group compared to the activated M1 macrophages that received no siRNA associated treatment (Fig. 3). At the longer 48 h incubation period, siRNA w/TR was able to decrease iNOS expression in LPS and IFNγ challenged M1 macrophages (***p ≤ 0.001). A significant downregulation of iNOS was also observed after only a 4 h pre-treatment of 10%Ab-Chi/siRNA NPs (***p < 0.001) and siRNA-chitosan NPs (**p < 0.01) (Fig. 3). Moreover, as the antibody density increased in the siRNA nanocomplexes, the ability to inhibit iNOS expression decreased. This effect may be caused by a lower charge density, which may have resulted in particle instability and poor macrophage uptake. Compared to untreated M1 macrophages responses, no differences in iNOS expression were observed using any of the scrambled siRNA complexes.

### Reduction of nitric oxide (NO)

**Fig. 4:**
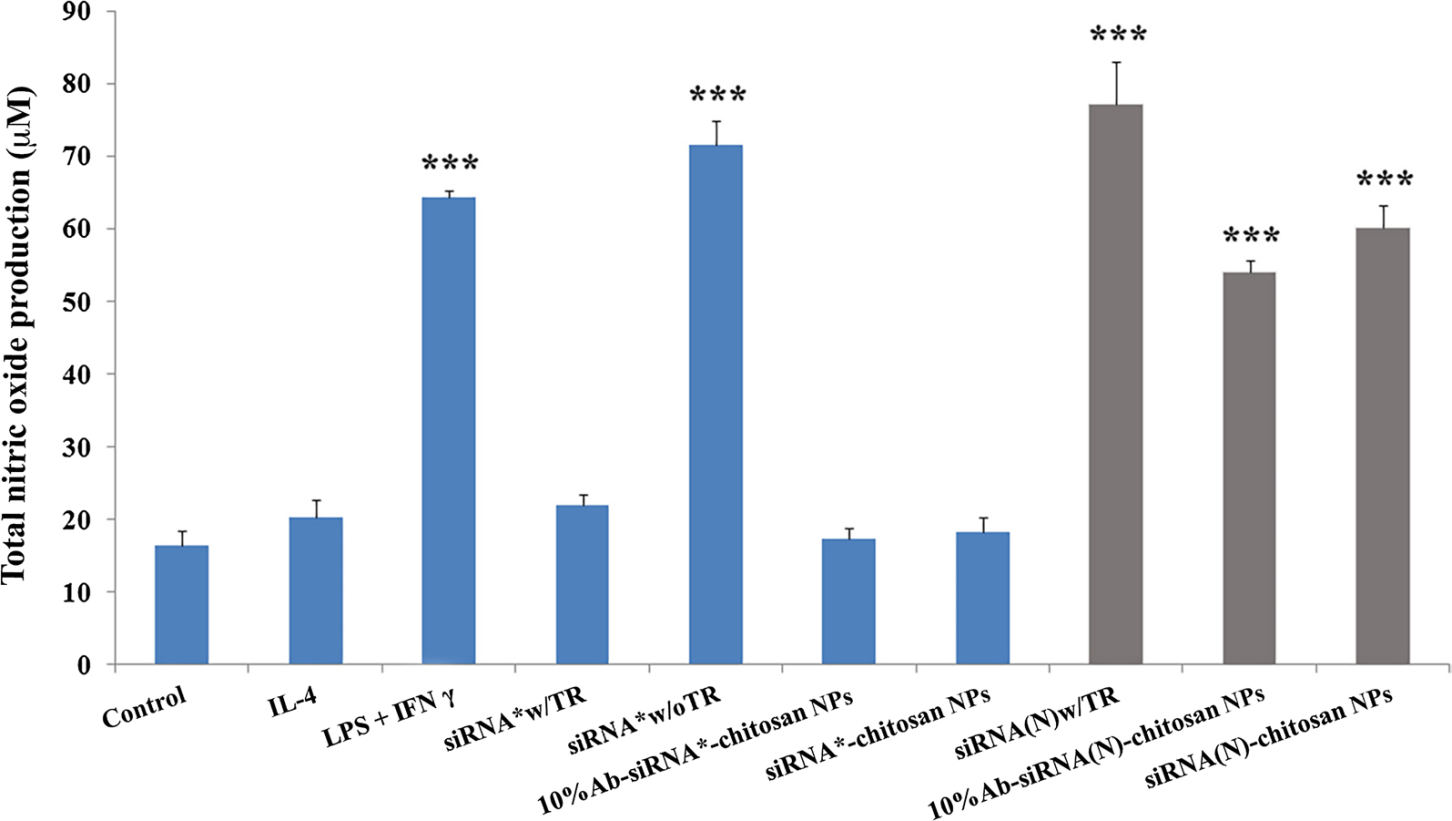
Downregulation of nitric oxide production after siRNA treatment by activated M1 macrophages. The groups are indicated as: no cytokine stimulation (control), M2 polarized macrophages (IL-4), M1 activated macrophages without siRNA treatment (LPS + IFNγ), naked siRNA delivered with transfection reagent for 48 h (siRNA* w/TR) or without transfection reagent (siRNA* w/o TR). siRNA was also coupled to chitosan NPs with (10% Ab-siRNA*-chitosan NPs) or without (siRNA*-chitosan NPs) antibody targeting moiety. Scrambled siRNA sequences were also used to screen for nitric oxide changes and these groups (*grey bars*) are denoted as siRNA(N). ***p ≤ 0.001 vs control

Nitric oxide, the main product of iNOS generation, is detrimental to and exacerbates the primary spinal cord injury. Figure 4 shows that all treatment groups significantly reduced NO level (***p < 0.001) compared to no treatment and corroborates the silencing efficiency of iNOS targeted siRNA (siRNA* in Fig. 3). We also quantified NO production for siRNA treatment without a transfection reagent. In these cases, increased NO production suggest poor cellular penetration of naked siRNA strands. To verify the specificity of the siRNA, we also exposed the macrophages to scrambled siRNA [siRNA(N)] sequences. Again, results showed that scrambled siRNAs did not affect NO generation with values comparable to no treatment controls (Fig. 4).

### Inhibition of cell apoptosis after spinal cord injury

**Fig. 5:**
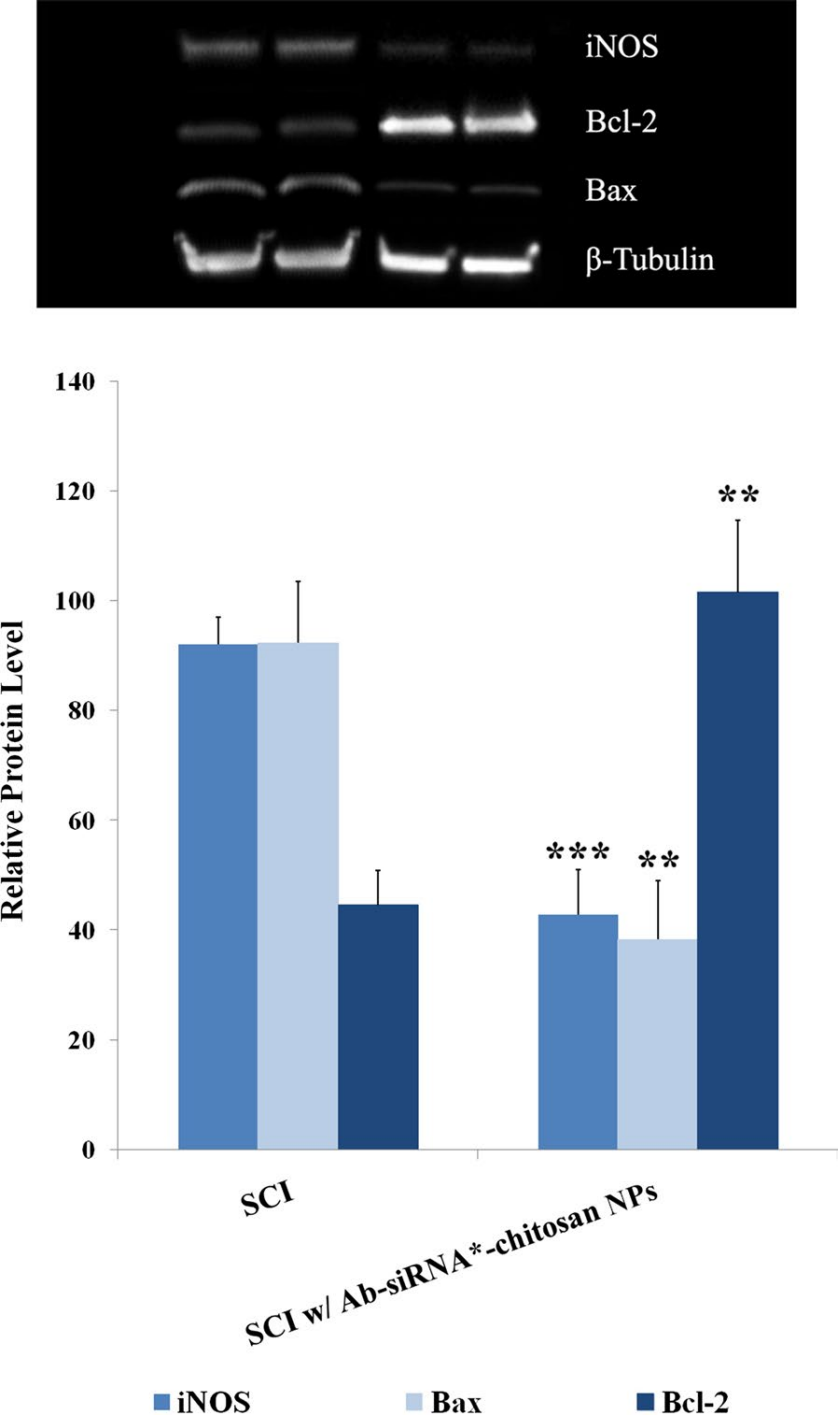
siRNA coupled chitosan Nps reduced levels of iNOS and apoptotic biomarkers at 24 h post-SCI. iNOS, Bcl-2 and Bax were measured in both sham animals and in mice treated with 10% Ab-siRNA-chitosan NPs. β-Tubulin served as the protein control. ***p ≤ 0.001, **p ≤ 0.01

iNOS, Bax and Bcl-2 protein expression were investigated using western blotting methods at 24 h after SCI (Fig. 5). In comparison to sham animals, the 10% Ab-siRNA-chitosan NPs treatment group showed approximately 50% reduction in both iNOS and pro-apoptotic Bax expression. This was accompanied by a near twofold increase in anti-apoptotic Bcl-2 protein levels (***p ≤ 0.001 for iNOS; **p ≤ 0.01 for Bax and Bcl-2). β-tubulin was used as the protein control.

## Discussion

Macrophages are critical cellular players following tissue injury and perform a variety of functions that include host tissue defense, phagocytosis of cells/debris, and secretion of cytokines. Macrophages are broadly characterized into two main classes, the classical pro-inflammatory (M1) group and the alternatively activated anti-inflammatory (M2) cohort. M1 macrophages have been reported to be the long-lasting residents at injury sites post-SCI and play a role in the propagation of neuroinflammation within the microenvironment of the spinal cord [6, 24, 25]. M1 macrophages can secrete TNF-α, and produce high levels of reactive oxygen and nitrogen species. These species have been implicated in demyelination and axonal degeneration following SCI [26]. A hallmark feature of M1 macrophages is the overexpression of iNOS and its downstream product, NO [6]. Previous immunohistochemical study has shown that iNOS positive cells peak at 24 h and declines to undetectable levels within 3 days after spinal cord injury [27]. To this end our goal was to disrupt iNOS overproduction in the acute stage of SCI using targeted siRNA. Since initial discovery, siRNA has been employed to regulate gene expression in a variety of disorders ranging from viral infections to immune disease and cancer [28, 29]. Multiple carriers have been engineered to deliver siRNA to target cells. These include viruses, lipid transfection, cyclodextrin, and dendrimers [30, 31]. In our case, we used chitosan as the vehicle due to its versatility and unique membrane fusogenic properties [23]. The chitosan was synthesized into NPs and formulated with IgG moieties to facilitate receptor mediated phagocytosis via the Fc receptors (FcRII/CD16 and/or FcRIII/32) expressed on M1 macrophages [6]. For vehicle synthesis, multiple parameters were considered [32]. For example, the molecular weight of chitosan, which is associated with particle size, was in the range of 65–170 kDa. This resulted in a particle size of 270–290 nm and was consistent to NPs from earlier studies using similar molecular weights [33, 34]. Also, the degree of deacetylation (DD) was varied. The DD denotes the percentage of deacetylated primary amine groups along the chitosan backbone, a determinant of charge density. We found that a DD value of 75–85% produced highly positively charged NPs, which enabled greater siRNA binding capacity and stability as demonstrated with gel electrophoresis. The weight ratio between chitosan and siRNA was chosen to be 37.5 based on prior results that reported a balanced toxicity and stability condition of chitosan materials during siRNA delivery [33]. IgG density was screened via particle size as chitosan NPs with a higher antibody ratio possessed larger particle diameters due to agglomeration and reduced charge density. Since low charged polymeric NPs have been shown to have an inefficient internalization rate for macrophages compared to a higher surface charged NPs [35], siRNA-chitosan NPs with 10% v/v antibody content (which also showed higher iNOS inhibition) was selected for further studies.

In vitro experiments were subsequently performed to verify macrophage polarization, NPs cytotoxicity/uptake, and iNOS silencing efficacy. Macrophages were first induced into the M1 state with LPS and IFNγ. These macrophages underwent a morphology change toward a more rounded or disc configuration, and iNOS expression was significantly upregulated. In contrast, macrophages stimulated with IL-4 were more spindle shaped. These M2 polarized cells did not produce detectable changes in iNOS. This observation corroborates other reports whereby macrophage morphology is associated with cell polarization and cytokine production [36]. Qualitatively, cytotoxicity studies using morphology showed chitosan NPs were well tolerated, whereas the lipid-based transfection agents caused noticeable cell death. The antibody coated NPs may have also improved phagocytosis, especially by M1 macrophages, which tend to exhibit higher phagocytic activity [37, 38]. When applied to M1 polarized macrophages, IgG coupled siRNA-chitosan NPs significantly suppressed both iNOS and NO production via Fc-receptor-mediated phagocytosis (note that multiple siRNA sequences were screened, see Additional file 1: Figures S3, S4). Interestingly, iNOS was also reduced in siRNA-chitosan NPs (w/o IgG) albeit with lower efficacy. In this instance, low molecular weight chitosan may be binding to mannose receptors, which have been shown to facilitate nanoparticle internalization [39]. Other receptors, such as galactose, lectin-based receptors, and lipoprotein receptors, may play a role as they have been also implicated in macrophage-targeted NPs delivery [40]. Nonetheless, at shorter treatment durations (4 h), both types of siRNA-chitosan NPs demonstrated significantly more effective transfection efficiency and iNOS suppression compared to lipid-based transfection. Some findings have suggested that chitosan can downregulate expression of proinflammatory cytokines in macrophages, upregulate anti-inflammatory cytokines, and favor M2 polarization [41, 42]. However, scrambled siRNA sequences coupled to chitosan NPs did not attenuate iNOS generation in our study. These results imply that the siRNA sequences and not the carrier are responsible for the amelioration of iNOS and NO.

Based on the in vitro data, we subsequently applied the Ab-siRNA-chitosan NPs to mice subjected to traumatic spinal cord injury. Briefly, mouse cord was compressed to approximately 10% of its original width as in previous experiments with other rodent models [23]. At 24 h post-injury, which is coincident with peak iNOS expression [27], we observed iNOS was downregulated by approximately 50% in treated mice compared to the sham group. Further, markers for cell apoptosis were also significantly altered. Pro-apoptotic Bax expression decreased while anti-apoptotic Bcl-2 protein levels were increased compared to injured control groups [43]. Bcl-2 is known to inhibit Bax-mediated apoptosis via formation of homo and heterodimers [44, 45]. This reversal in the Bax/Bcl-2 profile may have downstream effects in protecting cells from undergoing apoptosis. These results suggest the application of Ab-siRNA-chitosan NPs may have suppressed the M1 macrophage response, leading to a reduction in RNS generation and a sparing of bystander cells after SCI. However, it is unclear how these early changes in iNOS expression and apoptosis may translate into longer term functional outcomes. The cumulative data provides support that iNOS targeted siRNA combined with chitosan NPs may be a viable therapeutic approach following SCI. Future investigations will look into possible changes in histological, functional/behavior changes that may benefit from application of these NPs. Nonetheless, these preliminary results show that the chitosan NPs offer some neuroprotective effects in attenuating the onset of secondary injury.

## Conclusion

In conclusion, we introduce a novel siRNA based gene silencing strategy to reduce iNOS and NO production by targeting pro-inflammatory macrophages after SCI. We successfully synthesized and tested siRNA-chitosan NPs capable of delivering siRNA sequences at high transfection efficiency with low cytotoxicity to macrophages. These cationic complexes further show good stability with minimal agglomeration in solution. Proof of concept in vivo data in mice demonstrated significantly low iNOS expression and a reduction in apoptotic process. Experimental data provides a promising basis for continued work in siRNA mediated gene silencing to reduce the propagation of secondary injury after spinal cord trauma.

## Additional file

**Additional file 1.** Additional Figures S1 through S5.

## Abbreviations

siRNA: small interfering RNA
SCI: spinal cord injury
iNOS: inducible nitric oxide synthase
NO: nitric oxide
NPs: nanoparticles
h: hours
EDC: ethyl-3-[3-dimethylaminopropyl]carbodiimide hydrochloride
sulfo-NHS: *N*-hydroxysulfosuccinimide
PAA: poly(l-aspartic acid)
MW: molecular weight
DD: degree of deacetylation
PEI: polyethyleneimine
