# Supplementary material for: Targeted siRNA delivery reduces nitric oxide mediated cell death after spinal cord injury
Source: J Nanobiotechnology. 2017 May 8;15:38. doi: 10.1186/s12951-017-0272-7 (PMC5422875; doi:10.1186/s12951-017-0272-7)
Supplement: Supplementary file 1 — Additional file 1. Additional Figures S1 through S5. [file 12951_2017_272_MOESM1_ESM.docx]

**Additional file 1:**

**
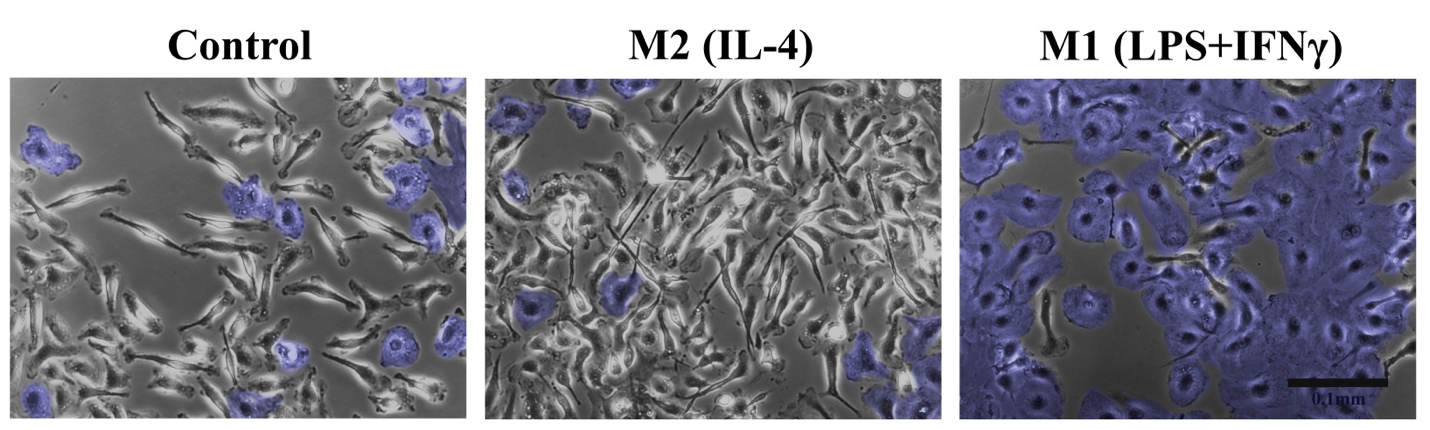
**

**S1.** The morphology of activated macrophages subsets. The control group did not receive any cytokine stimulation. Macrophages activated with IL-4 were polarized into the M2 stave, whereas macrophages receiving LPS and IFNγ were activated into the M1 state. M2 and control macrophages demonstrated a spindle-like morphology while M1 macrophages were more spread out and rounded in appearance (shaded in blue).


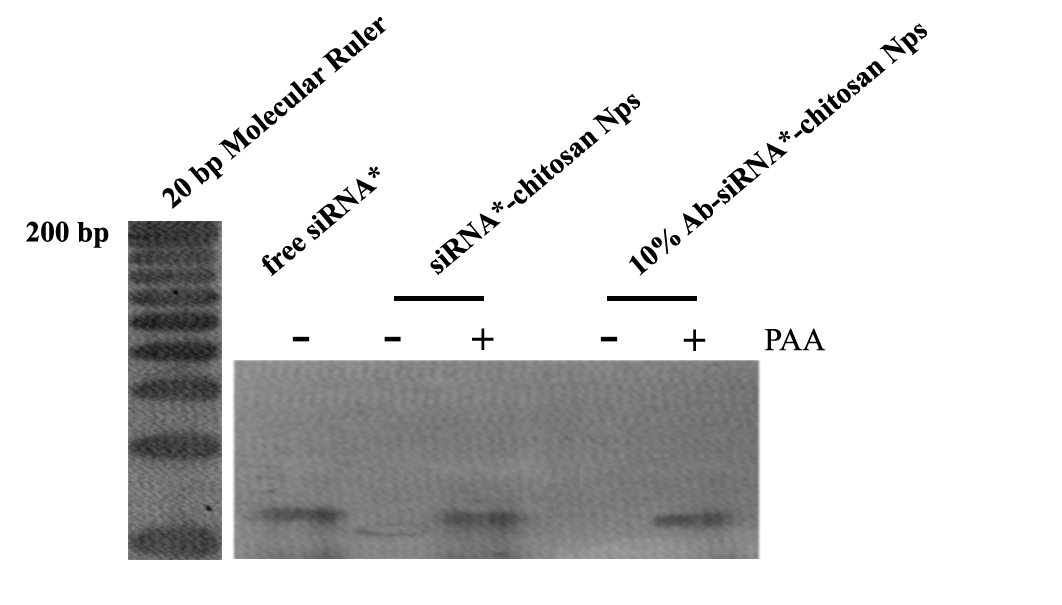


**S2.** Gel retardation assay indicating the integrity of chitosan nanoparticles. Poly(l-aspartic acid) (PAA) is a highly anionic polyelectrolyte that competitively binds to cationic chitosan, displacing siRNA.


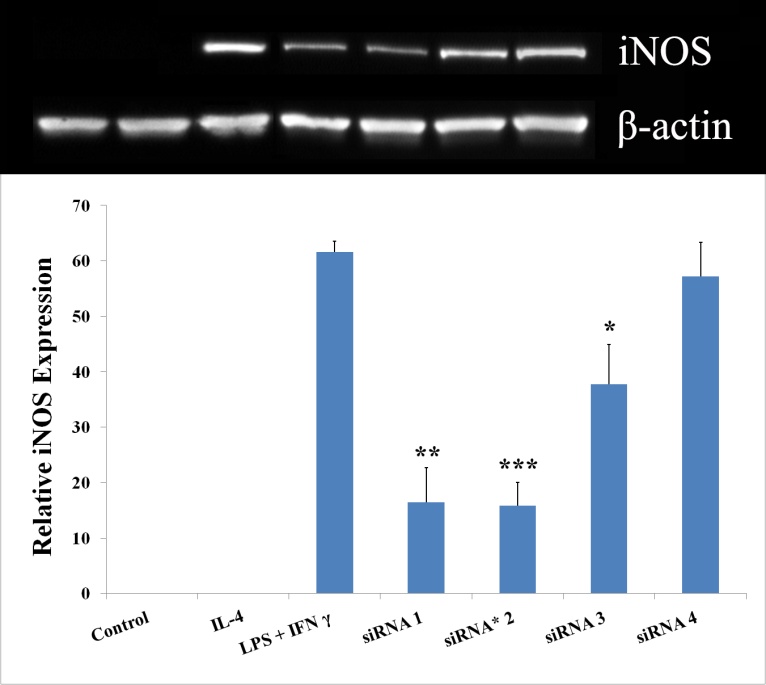


**S3.** Screening of four unique iNOS targeted siRNA sequences (siRNA1-siRNA4) to detect the silencing effects on iNOS mRNA expression using Western blotting techniques. **p< 0.01 and ***p<0.001 vs LPS+ IFNγ.

**
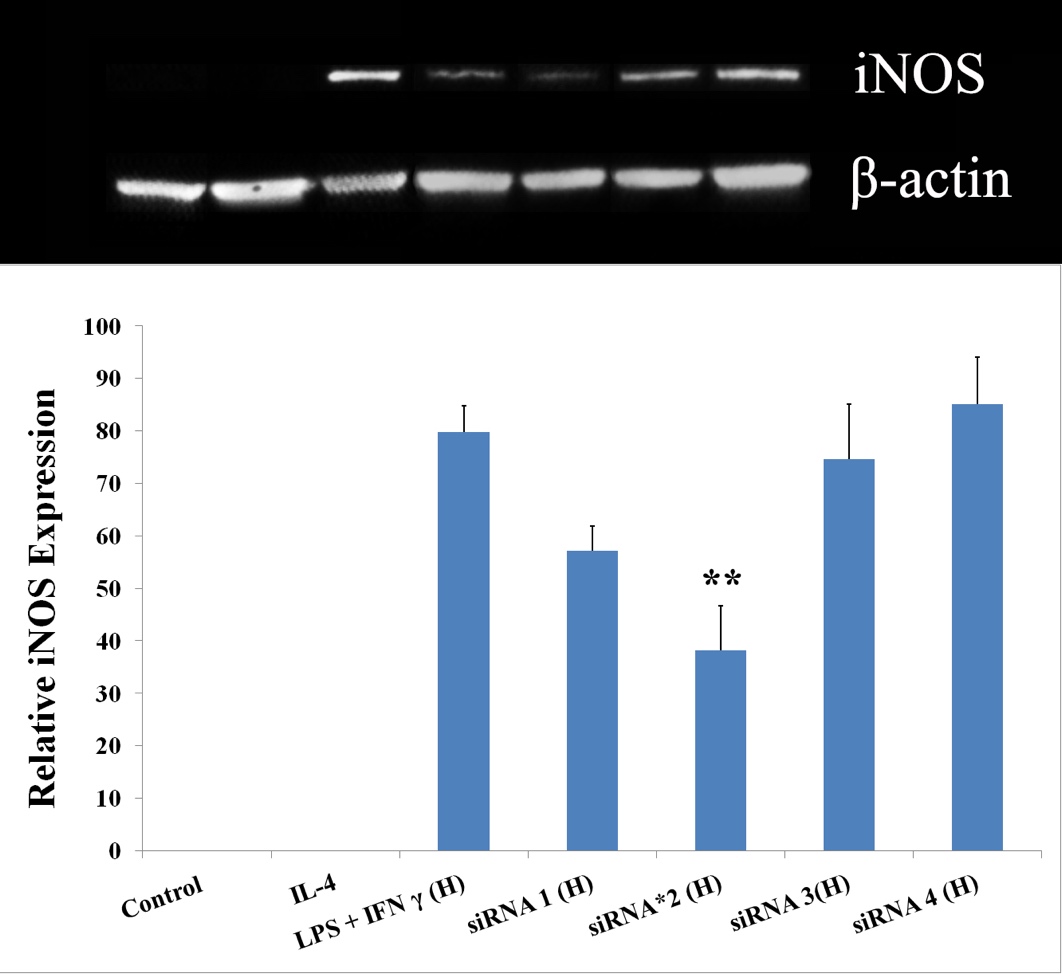
**

**S4.** Screening of four unique siRNA sequences to detect the silencing effects on iNOS mRNA expression when a high concentration of 1μg/ml LPS +200ng/ml IFNγ is applied to activate macrophages into the M1 state. **p< 0.01 vs LPS+ IFNγ.


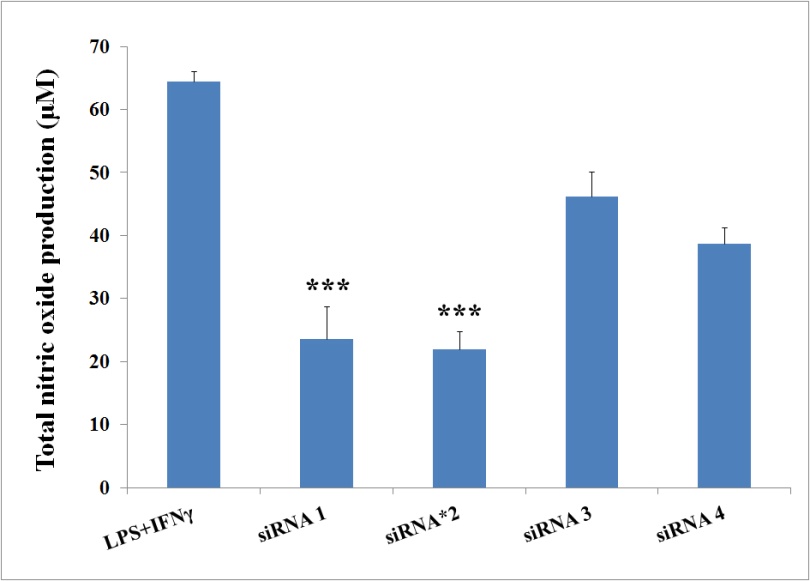


**S5.**  Screening and comparison of nitric oxide secretion from BMDM with and without siRNA treatment. ***p<0.001 vs LPS+ IFNγ.
